# Toward practical BCIs: a BMNABC-based feature selection and sensor optimization framework for implicit learning detection from multimodal EEG-fNIRS data

**DOI:** 10.3389/fnhum.2026.1778884

**Published:** 2026-05-04

**Authors:** Chayapol Chaiyanan, Tustanah Phukhachee, Keiji Iramina, Boonserm Kaewkamnerdpong

**Affiliations:** 1 Department of Computer Engineering, Faculty of Engineering, King Mongkut’s University of Technology Thonburi (KMUTT), Bangkok, Thailand; 2 Graduate School of Systems Life Sciences, Kyushu University, Fukuoka, Japan; 3 Biological Engineering Program, Faculty of Engineering, King Mongkut’s University of Technology Thonburi (KMUTT), Bangkok, Thailand

**Keywords:** education neuroscience, electroencephalography, feature selection, functional near-infrared spectroscopy, implicit learning, multimodal neuroimaging, sensor optimization

## Abstract

Implicit learning is a fundamental cognitive process whose identification is critical for understanding human cognition and developing innovative training methodologies. We propose a generalizable feature selection and sensor optimization framework using simultaneous EEG and fNIRS to identify these events. Our approach leverages a two-stage optimization process driven by a binary multi-neighbor artificial bee colony (BMNABC) algorithm. The BMNABC uses the model’s classification accuracy to guide the heuristic search for the most discriminative feature subset. First, the framework prioritizes optimal features from high-dimensional, multimodal data using a normalized weighted sum (NWS) metric. Second, it implements a recursive backward elimination mechanism to reduce the number of sensors for practical brain-computer interface (BCIs) applications. Our results demonstrate that the BMNABC framework successfully identifies a superior feature set, leading to a significant improvement in classification accuracy over using either modality alone. Critically, the selected features provided neurophysiological validation, isolating key biomarkers in the prefrontal cortex. We also show that a sparse yet highly effective sensor configuration can be achieved, maintaining high performance with up to 66% fewer sensors. This work not only provides a data-driven method for detecting implicit learning but also advances the design of more efficient and user-friendly BCI systems.

## 1 Introduction

In our rapidly evolving world, characterized by continuous innovation and technological advancements, the ability to rapidly acquire and integrate new knowledge is crucial for maintaining competitiveness and fostering progress. Traditional explicit learning methods, often relying on structured instruction and formal assessments, may no longer be sufficiently agile to meet the demands of this accelerated pace. Implicit learning, which allows for knowledge acquisition without conscious awareness or direct instruction (Seel, 2012), offers a promising alternative for accelerating the learning process. This form of learning is exemplified by natural language acquisition in early childhood or the development of motor skills like riding a bicycle, where knowledge is gained gradually and without explicit instruction or the learner being able to pinpoint when the learning has occurred.

The benefits of proficiency in implicit learning are substantial. Implicit learning techniques are frequently employed deliberately in educational settings, such as through educational games or television programs, to facilitate intuitive and engaging material acquisition. Previous research indicates that individuals who excel at implicit learning can more effortlessly acquire other skills requiring similar cognitive abilities (Frensch and Runger, 2003). Furthermore, technology can enhance implicit learning; its asynchronous nature, for instance, often encourages learners to interpret nuances and ‘read between the lines,’ fostering deeper implicit understanding (Marsick and Watkins, 2001). Despite this significant potential, the precise neural mechanisms underpinning implicit learning and the reliable identification of implicit learning events remain considerable challenges. Our study is motivated by the need to bridge this gap, aiming to develop robust methods for identifying implicit learning events, thereby contributing to a deeper understanding of this vital cognitive process and its practical applications.

Attempts have been made to identify the emergence of implicit learning using neuroimaging techniques. For instance, Rose et al. (2010) conducted an experiment where participants implicitly learned a complex rule while solving a cognitive problem. They introduced a behavioral marker, the triple response time (TRT), to signify the emergence of learning. Concurrently, participants’ brain activities were recorded using EEG and fMRI. Rose et al. observed increased neural activity and high-frequency coupling in distant brain areas preceding the TRT, suggesting that distinct neurobiological features can be identified around these learning events. However, a significant limitation of their approach for broader application is the reliance on fMRI. While powerful, fMRI machines are expensive, cumbersome, and unsuitable for routine or everyday use. This highlights the need for more accessible and practical neuroimaging alternatives for identifying implicit learning.

Unlike neuroimaging modalities such as fMRI and PET, electroencephalography (EEG) and functional near-infrared spectroscopy (fNIRS) offer significant advantages for studying brain activity, especially in less constrained environments. Both are non-invasive, portable, relatively affordable, and safe for long-term monitoring (Lenkov et al., 2013; Machado et al., 2011). While EEG provides exceptional temporal resolution for capturing rapid electrical brain activity (Michel and Murray, 2012), it traditionally suffers from poor spatial resolution and susceptibility to motion artifacts (Kus et al., 2004; Wessel et al., 2011; Blinowska, 2011; Babiloni et al., 2009). Conversely, fNIRS measures cerebral hemodynamics (Sassaroli and Fantini, 2004), offering a better balance of spatial and temporal resolution with high mobility. The integration of these two modalities leverages their complementary strengths to mitigate individual limitations, providing a more robust and comprehensive understanding of brain function during complex cognitive processes like implicit learning.

Research has consistently demonstrated the benefits of integrating these two modalities, particularly in brain-computer interface (BCI) applications. For example, Fazli et al. (2012) proposed a hybrid sensory motor rhythm-based BCI model that combined fNIRS and EEG signals, achieving an average improvement of +5% in motor imagery classification accuracy. Similarly, Khan et al. (2014) successfully decoded four movement directions by combining fNIRS and EEG features, and Koo et al. (2015) developed a novel hybrid BCI system for online self-paced motor imagery by using fNIRS to detect motor imagery occurrence and EEG for classification.

Further studies reinforce the advantages of this bimodal approach. Yin et al. (2015) reported a 1–5% improvement in classification accuracy for a motor imagery task when combining fNIRS and EEG features compared to using either modality alone. Putze et al. (2014) demonstrated the efficiency of concatenated features from bimodal EEG-fNIRS in improving BCI versatility for auditory and visual tasks. Even in clinical applications, Blokland et al. (2014) found improved accuracy in brain switch control for patients with tetraplegia when combining these modalities. Recent studies have further validated this, with multimodal frameworks that utilize CNN-LSTM architectures (Ehsan et al., 2022) or multi-domain feature sets (Subasi and Qaisar, 2021) achieving classification accuracies significantly higher than those of unimodal approaches. The synergy of EEG-fNIRS technology extends to various fields, including language studies, cortical current estimation, and fatigue monitoring (Wallois et al., 2012; Morioka et al., 2014; Ahn et al., 2016; Calhoun et al., 2006).

By fusing these complementary signals at the feature level, their respective shortcomings in spatial and temporal resolution can be overcome, leading to hypothesized improvements in classification accuracy over individual signal results. However, this multimodal integration inherently introduces significant challenges. The combination of high-dimensional data from both EEG and fNIRS sources results in a substantially larger and more complex feature space. This increased dimensionality often contains redundant and irrelevant features, which can obscure meaningful patterns, introduce noise, and consequently diminish the performance and interpretability of subsequent classification models. Therefore, effectively identifying implicit learning events from such complex datasets becomes considerably more difficult without a robust mechanism to manage this high dimensionality. This necessitates the application of sophisticated feature selection techniques to identify the most salient and discriminative information. The need for such precision mirrors recent trends in other engineering domains, where data-driven parameter estimation—such as the use of artificial neural networks to identify critical electrical variables in PMDC motors (Siddiqi et al., 2024)—has become essential for modeling non-linear system behaviors with high integrity.

The objective of this study is to develop and validate a feature selection framework for identifying implicit learning events from simultaneously acquired EEG-fNIRS data. Building upon the challenges of high-dimensional multimodal data, we aim to demonstrate the efficacy of advanced feature selection and classification techniques for precise event detection. We further investigate the impact of reducing the number of selected electrodes and optodes on classification accuracy, thereby demonstrating the potential for a more streamlined and practical system suitable for BCI applications. Achieving this objective will not only deepen our understanding of implicit learning but also support the development of practical BCIs. This study builds upon our previous finding that the artificial bee colony (ABC) algorithm effectively identifies implicit learning events from EEG entropy features (Chaiyanan et al., 2021). By extending this to a hybrid system with sensor optimization, we aim to meet the current demand for modular, wireless neuroimaging technologies deployable in naturalistic environments (Uchitel et al., 2021).

## 2 Materials and methods

This section details the methodologies employed in this study, outlining the comprehensive pipeline from data acquisition to the final feature selection and classification. We begin by describing the simultaneously acquired EEG-fNIRS dataset, which forms the foundation of our analysis. Subsequently, the essential pre-processing steps applied to both neuroimaging modalities are presented, ensuring data quality and readiness for feature extraction. The core of our analytical approach involves distinct feature extraction techniques for each modality: Entropy and Multiscale Entropy (MSE) are utilized to capture the complexity of EEG signals, while Wavelet Transform is applied to extract relevant features from fNIRS data. Finally, we detail our novel ABC based feature selection framework, which is designed to identify the most discriminative features from the combined multimodal dataset to accurately classify implicit learning events.

### 2.1 Simultaneous EEG-fNIRS dataset

For this study, we utilized a dataset previously collected to investigate methods for classifying implicit learning events (Chaiyanan et al., 2021; Chaiyanan et al., 2025). This dataset comprises simultaneous EEG and fNIRS recordings from 30 college students (aged 21–29), none of whom had a diagnosed learning disability. The sensors were arranged in a hybrid montage where EEG electrodes and fNIRS optodes were co-located at identical scalp positions according to the international 10–20 system. The study protocol was approved by the Experimental Ethics Committee of the Faculty of Information Science and Electrical Engineering, Kyushu University (ISEE H26-3, 23 June 2014), and all participants provided written informed consent prior to the experiment in accordance with ethical guidelines for human research. During the experiment, participants engaged in a cognitive task without explicit instructions on how to solve the problems. Participants were presented with a series of question sets and asked to provide answers by pressing a button. Each set comprised four questions, constituting a single trial. Up to 180 trials were completed per participant while their neurological data was recorded.

Two primary rules governed all the questions in each trial. The fourth question, however, was subject to an additional, implicit rule: its answer was always identical to that of the first question in the same trial. Participants received negative auditory feedback (a beep) for incorrect answers related to the two primary rules, but this feedback offered no indication regarding the third, implicit rule. Achieving implicit learning was defined by the participant’s acquisition of this hidden third rule.

Implicit learning was primarily identified by analyzing response times (RTs). A consistent reduction in the RT for the fourth question, relative to the other questions within the same trial, served as a key behavioral indicator that implicit learning had occurred. To further validate this, post-experiment interviews were conducted. While all participants could easily articulate the first two explicit rules, only 9 out of the 30 participants were able to verbalize the additional rule governing the fourth question. These nine individuals were subsequently categorized as having successfully achieved implicit learning.

### 2.2 Data preprocessing

We preprocessed EEG data to ensure optimal quality for feature extraction. EEG data underwent several critical steps. First, we applied a bandpass filter between 0.5 and 50 Hz to isolate relevant brainwave frequencies. A 60 Hz Notch filter was then used to effectively remove power line noise. To further eliminate non-brain source artifacts, such as muscle movements or eye blinks, we employed independent component analysis (ICA), a robust blind source separation method (Mahajan and Morshed, 2015). For fNIRS data, we did not apply separate preprocessing filters. Instead, the chosen feature extraction method, as detailed in the subsequent section, was specifically selected to inherently handle any noise associated with the fNIRS signals. The continuous time-series EEG and fNIRS data were segmented into epochs for each trial.

Beyond neurophysiological data, we analyzed response times (RTs) for each trial. To mitigate the influence of outliers, a moving median filter with a window of 5 was applied to the RT data. The response time for the fourth question in each trial was designated as the determined response time (DRT), while the average response time of the remaining questions in that same trial was labeled as the undetermined response time (URT). To define implicit learning events objectively, each trial was categorized using a subject-specific 95% confidence interval (CI) derived from the participant’s URT distribution. A trial was labeled ‘Fast’ if the DRT fell below the lower bound of this 95% CI, indicating a statistically significant behavioral acceleration characteristic of an implicit learning event; otherwise, it was labeled ‘Slow’.

The validity of this labeling strategy is supported by a Wilcoxon Signed-Rank Test applied to the behavioral data previously reported in our prior investigation (Chaiyanan et al., 2025). For the ‘Yes Implicit Learning’ group (*n* = 9), the test confirmed a significant reduction in RT during the post-learning phase, with the mean DRT for implicit rules (666.7 ± 155.3 ms) being substantially lower than the mean URT for explicit rules (1027.9 ± 99.8 ms, *p* = 0.0039). No such significant difference was found in the pre-learning phase for the same group (*p* = 0.4961). Conversely, the ‘No Implicit Learning’ group (*n* = 21) showed no statistically significant divergence in RT between trial types across the entire experiment (*p* = 0.1315). This subject-adaptive approach was chosen specifically to mitigate subject-specific signatures by normalizing the threshold to each individual’s cognitive baseline speed. This ensures the model identifies the relative shift in neural state associated with learning the hidden rule rather than baseline behavioral speed. While subject-level validation (verbalization of the rule by 9 out of 30 participants) confirms the final acquisition of explicit knowledge, the trial-level ‘Fast’ labels are designed to capture the emerging neural signatures of implicit pattern recognition that precede conscious awareness.

Given that implicit learning is a time-sensitive process, maintaining the chronological integrity of the hemodynamic and electrophysiological signals was prioritized over total participant volume. From the initial pool of 30 participants, a high-integrity subset of 10 individuals (5 meeting the implicit learning criteria and 5 non-implicit learners) was selected for the optimization framework. The selection is based on signal-quality, artifact-free recording sessions without temporal discontinuities. This resulted in a balanced dataset of 350 trials (175 ‘Fast’ and 175 ‘Slow’), providing a robust and non-biased foundation for the BMNABC optimization process.

### 2.3 EEG feature extraction using entropy and multiscale entropy

Human physiological signals, particularly electroencephalogram (EEG) signals, are inherently complex, non-linear, and susceptible to noise interference. Traditional linear methods often fall short of adequately characterizing this complexity. To overcome this, the concept of entropy, initially derived from thermodynamics to quantify system randomness (Clausius, 1879; Borowska, 2015), was extended by Shannon to information theory for measuring system complexity or informational content (Shannon, 1948). This concept has since found significant application in analyzing complex biomedical signals (Bao et al., 2018; Wang et al., 2017; Peng et al., 2017). For instance, Pincus (1991) successfully employed approximate entropy (ApEn) to analyze heart rate variability in infants, demonstrating its potential in the early detection of sudden illness; ApEn proved particularly suitable for biomedical signals, often characterized by short, noisy segments. Building upon this work, Richman and Moorman developed Sample Entropy (SampEn), an enhancement designed to perform more effectively with data of varying lengths, a common characteristic of biomedical data (Richman and Moorman, 2000; Yan et al., 2012). These entropy-based methods are thus particularly valuable for characterizing the fluctuating and erratic nature of EEG.

In this research, Sample Entropy (SampEn) was selected for quantifying EEG signal complexity due to its robustness and suitability for relatively short and noisy biomedical data segments (Liang et al., 2015). SampEn measures the predictability of a time series, with higher values indicating greater complexity and unpredictability. To compute SampEn, continuous EEG data was first divided into short sequences or epochs. For each epoch, SampEn was calculated as defined by:


$$\text{SampEn}(m,r,N)=-\log\frac{\sum A_{i}}{\sum B_{i}}=-\log\frac{A}{B}$$

where m denotes the template length, r represents the tolerance for accepting matches, and N is the number of data points within each epoch. $A_{i}$ signifies the count of forward matches of length m+1 , while $B_{i}$ represents the count of matches of length m. A SampEn value of zero indicates a complete absence of complexity within the data, occurring when the number of matches for both template lengths m and m+1 are equivalent. Conversely, a smaller number of matches for the longer template (m + 1), which is inherently expected as the template length increases, results in a higher SampEn value, reflecting greater data complexity. Fluctuations in EEG signal complexity, indicative of changes in brain activity, are directly reflected in corresponding SampEn values.

To address the limitations of analyzing complexity at a single time scale, Multiscale Entropy (MSE) was employed. MSE extends SampEn by evaluating data complexity across multiple temporal scales through a coarse-graining procedure (Catarino et al., 2011; Wu et al., 2013; Morabito et al., 2012). This process involves successively averaging data points within an epoch at different scales, allowing for the quantification of complexity across a range of neural dynamics. For our analysis, an optimal template length (m) of 2 and a tolerance (r) of 0.15 times the standard deviation of the epoch were applied. These specific parameter choices are grounded in established physiological time-series analysis protocols, where m = 2 and r values between 0.1 and 0.2 have been shown to provide optimal statistical power and consistency for relatively short biomedical data segments (Richman and Moorman, 2000; Yan et al., 2012; Wu et al., 2013). With a sampling frequency of 1,000 Hz and a maximum epoch length of 3 s, a maximum scale (*τ*) of 20 was utilized, ensuring the recommended minimum number of data points (*N* = 150) per scale for reliable SampEn estimation (Richman and Moorman, 2000). The resulting SampEn values across multiple scales serve as robust features representing the dynamic complexity of EEG signals within each trial. Recent BCI research confirms that incorporating multiscale features is highly effective for extracting the hidden, non-linear discriminative patterns necessary for high-accuracy motor imagery and cognitive task classification (Subasi and Qaisar, 2021).

### 2.4 fNIRS feature extraction using wavelet transform

Functional Near-Infrared Spectroscopy (fNIRS) data were acquired using a Hitachi ETG-7100 system (Hitachi, Tokyo, Japan) at a sampling frequency of 10 Hz. For each trial, the analysis focused on changes in oxygenated hemoglobin (HbO2) concentration, determined by the modified Beer–Lambert law. Consistent with the EEG data, fNIRS data were also segmented into epochs based on trial and question markers.

The Wavelet transform (WT) was employed for both the preprocessing and feature extraction of the fNIRS data, as it provides a multi-resolution analysis particularly suited for the non-stationary nature of hemodynamic signals. WT enables the decomposition of signals into approximation functions and corresponding detail coefficients across various decomposition levels, effectively separating noise from relevant physiological responses. In this study, the Daubechies-8 (db8) wavelet family was selected because its mathematical properties and shape closely approximate the canonical hemodynamic response function (HRF), facilitating the precise isolation of task-related oxygenation changes from non-stationary noise (Molavi and Dumont, 2012). The selection of db8 and the optimal decomposition levels (L5 and L6) was empirically determined based on their capacity to yield the highest energy for trials categorized as “Fast,” and further validated by evaluating the ratio between the energy and the entropy of the reconstructed signals at these levels, a methodology that has shown promising results in similar signal analysis applications (Yan and Gao, 2009). The detail coefficients derived from these selected levels (L5 and L6) then served as the extracted features for the fNIRS data.

By focusing on levels L5 and L6, the framework effectively implements a multi-resolution filter that isolates task-related hemodynamics (approximately 0.07–0.31 Hz). This ensures that while the most discriminative learning markers are preserved, higher-frequency physiological interference (such as heart rate) and low-frequency baseline drifts are inherently filtered out. For this study, oxygenated hemoglobin (HbO2) was prioritized due to its documented superior sensitivity to task-evoked changes and a significantly higher signal-to-noise ratio compared to deoxygenated hemoglobin in multimodal BCI tasks (Putze et al., 2014; Kinder et al., 2022).

### 2.5 Feature selection framework using the binary multi-neighbor artificial bee colony (BMNABC) algorithm

The fusion of EEG and fNIRS data, while offering comprehensive insights, generates a high-dimensional feature space that can compromise model efficiency and performance. Effective feature selection is therefore critical to identify the most relevant and non-redundant features, enhancing classification accuracy and model interpretability for implicit learning event detection. For this task, we propose a framework based on the ABC algorithm. The ABC algorithm (Karaboga, 2005) is an empirically derived optimization technique inspired by the intelligent foraging behavior of honeybee swarms. Compared to other population-based metaheuristics like Ant Colony Optimization or Particle Swarm Optimization, ABC distinguishes itself by its relative ease of implementation due to a reduced number of control parameters. Furthermore, ABC incorporates a unique solution update mechanism that enhances its capacity to escape local optima and efficiently converge toward the global optimum, making it well-suited for complex feature selection problems.

Natural honeybee colonies exhibit a sophisticated division of labor and a robust communication system for disseminating information about nectar sources within the hive. Analogously, the ABC algorithm categorizes artificial bees into three distinct roles: employed bees, onlooker bees, and scout bees. Employed bees are responsible for exploiting current food sources and subsequently sharing the quality of these sources with the onlooker bees within the hive. Onlooker bees then probabilistically select food sources to exploit based on the information received from the employed bees. Scout bees undertake the exploration of the search space to discover new potential food sources. The decision regarding which food source a scout bee will investigate is influenced by the overall number and quality of existing food sources.

In the ABC framework, each food source represents a candidate solution to the optimization problem under consideration—in this case, a specific subset of features. The quality or fitness of a food source at a particular location within the search space is evaluated directly by the classification accuracy achieved when a model is built using that specific subset of features. This classification accuracy, serving as the fitness value, guides the collective foraging behavior of the ABC to identify the optimal feature subset that best discriminates implicit learning events. Ultimately, this process aligns with the core objective of feature selection, a critical machine learning technique aimed at identifying the most pertinent features while eliminating redundant or irrelevant ones. In contrast to feature extraction, which typically transforms original features, feature selection seeks an optimal subset of original features that maximizes classifier performance (e.g., accuracy) while minimizing error and information loss (Mahajan and Morshed, 2015).

**Figure 1:**
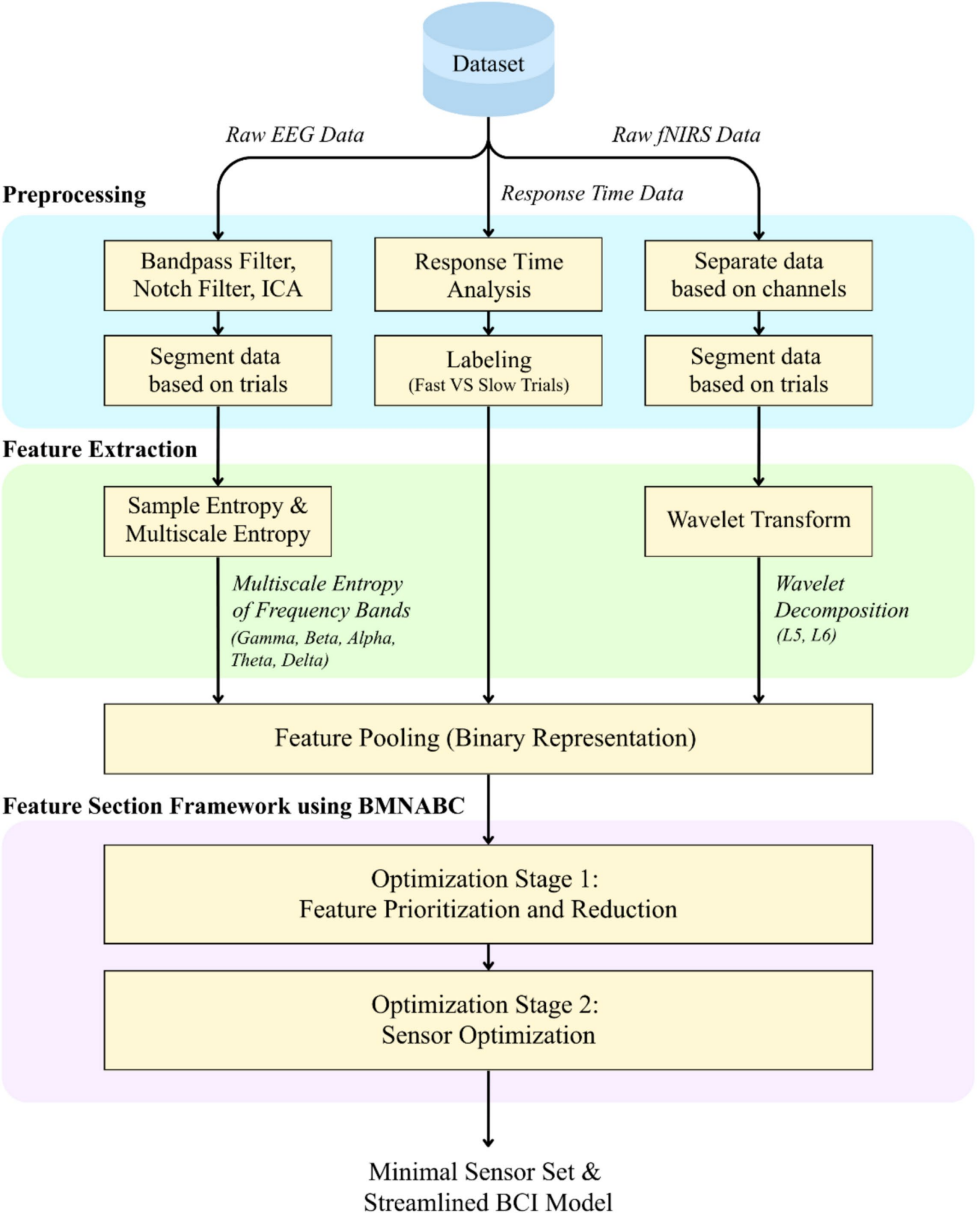
The comprehensive methodological framework proposed in this study. The pipeline illustrates the processing flow from simultaneous EEG-fNIRS data acquisition and behavioral labeling to the final output of a streamlined BCI model. The process encompasses data preprocessing, specific feature extraction methods (Entropy for EEG, Wavelet for fNIRS), and the novel BMNABC-based framework, which optimizes the system in two stages: first by reducing feature dimensionality, and second by minimizing the physical sensor configuration.

The proposed feature selection framework, leveraging the ABC algorithm, is visually represented in Figure 1. In this framework, each “food source” within the bee colony directly corresponds to a candidate subset of features, which can map to unique EEG electrode locations combined with specific frequency bands and to fNIRS wavelet reconstructed data. The core of the optimization process involves the binary multi-neighbor artificial bee colony (BMNABC) algorithm, which identifies optimal feature combinations. While other evolutionary strategies, such as Genetic Algorithms or Atomic Search Optimization, have been successfully applied to hybrid BCI channel selection (Rocha-Herrera et al., 2022; Qiu et al., 2022), BMNABC provides a strategic advantage in discrete binary spaces by balancing global exploration with local exploitation through its multi-neighbor search mechanism. For each selected set of features, a classification model is constructed, and its classification accuracy then serves as the fitness value guiding the BMNABC algorithm’s search. Furthermore, the framework incorporates a unique mechanism for iteratively removing features based on their contribution to classifier performance, aiming to reduce model complexity and enable the development of highly streamlined systems suitable for future BCI applications. The detailed components of this framework, including the feature representation, the BMNABC algorithm, and the feature reduction process, will be elaborated in the following subsections (2.5.1–2.5.3).

#### 2.5.1 Feature representation and encoding

For this study, the features considered for selection were derived from both EEG and fNIRS modalities, which were acquired simultaneously from co-located channels throughout the experiment. For EEG data, features were extracted from specific frontal channels (AF3, AF4, F3, F4, F5, F6, F7, F8, and FZ). This channel pre-selection was based on the established role of the frontal lobe in higher-order cognitive functions, including personality expression, complex planning, memory, and decision-making (Chizi et al., 2009). The raw EEG data from these channels were then filtered into Gamma, Beta, Alpha, Delta, and Theta frequency bands using entropy-based methods. For fNIRS data, the reconstructed signals from wavelet decomposition levels L5 and L6 constituted the initial feature set. The fNIRS optodes were integrated into the same EEG cap, with source-detector pairs fixed at a 3-cm distance and centered over EEG 10–20 coordinates to ensure the hemodynamic data precisely mirrored the electrophysiological recording sites.

**Figure 2:**
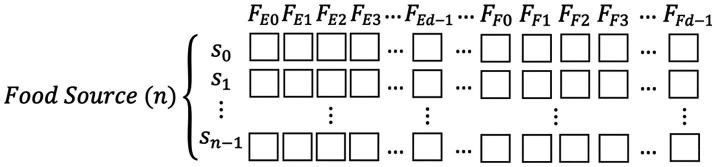
Binary encoding of the feature set as a ‘Food Source’ for the BMNABC algorithm. The diagram illustrates how individual features from EEG (*F_E0_* to *F_Ed-1_*) and fNIRS (*F_F0_* to *F_Fd-1_*) are represented as bits (1 for selected, 0 for excluded) within a single candidate solution.

During the feature selection process, each potential individual feature (e.g., a specific frequency band from an EEG channel, or a reconstructed fNIRS signal at level L5 or L6) was treated as a candidate. To represent the inclusion or exclusion of these features for the BMNABC algorithm, a binary encoding scheme was employed. A value of ‘1’ signified that a particular feature was selected for inclusion in the feature subset, while ‘0’ indicated its exclusion. These binary strings, representing distinct combinations of selected features, served as the ‘Food Sources’ within the BMNABC algorithm. Figure 2 illustrates the schematic setup of these feature sets, where *F_E0_* to *F_Ed-1_* represent features associated with EEG data, and *F_F0_* to *F_Fd-1_* represent features associated with fNIRS data. Each food source *S* thus explicitly encodes a unique feature subset to be evaluated for its classification performance.

#### 2.5.2 Binary multi-neighbor artificial bee colony (BMNABC) algorithm

The ABC algorithm, while offering several advantages, exhibits limitations in exploitation and can suffer from slow convergence in certain optimization scenarios (Zhu and Kwong, 2010). Notably, the standard ABC algorithm is not inherently designed for search operations within discrete binary spaces, which is a requirement for our feature selection problem. To address this, a specialized binary variant, the Binary Multi-Neighbor ABC (BMNABC), was developed (Beheshti, 2018). BMNABC distinguishes itself from the standard ABC by incorporating a strategic multi-neighbor search, utilizing information from both proximate and distant neighbors. Furthermore, instead of using a conventional rounding method, BMNABC employs a distinct transfer function to map solutions from a continuous real space to our discrete binary feature space.

The fundamental workflow of BMNABC mirrors that of the original ABC algorithm, but the search process for new food source positions in the employed bee and onlooker bee phases is specifically modified to enhance exploration and exploitation. During the employed bee phase, a bee is tasked with global exploration. It discovers a new food source position by leveraging information from a far neighbor, which is a strategic modification designed to encourage searching new, unexplored areas of the search space. Conversely, the Onlooker Bee phase focuses on local exploitation, with a bee updating its food source position based on the information from its best near neighbor. This strategic division of labor means the algorithm maintains a balance between discovering new areas and refining promising solutions.

The BMNABC algorithm was implemented following the architecture proposed by Beheshti (2018), specifically adapted for high-dimensional feature selection. To ensure reproducibility, the algorithm was initialized with a population of *n* = 20 bees and a maximum number of iterations of *N* = 100. The core of the BMNABC search process is the strategic use of neighborhood information determined by the Hamming distance between food sources.

During the employed bee phase, global exploration is facilitated by a search equation that leverages the average personal best position (*APB_ij_*) of a bee’s far neighborhood:


$$\begin{array}{c} v_{\mathrm{ij}}(t+1)=x_{\mathrm{ij}}(t)+\varphi (x_{\mathrm{ij}}(t)-\mathrm{AP}B_{\mathrm{ij}})\\ i\in \{0,1,\ldots ,n-1\},j\in \{0,1,\ldots ,d-1\} \end{array}$$

where *APB_ij_* is the average personal best position of the far neighborhood of the i^th^ bee in the j^th^ dimension, and φ is a random value in the range [0, 1]. *APB_ij_* was computed using the following equation


$$\begin{array}{c} \mathrm{AP}B_{\mathrm{ij}}=\frac{\sum_{k=1}^{\text{Nneigh}}\mathrm{rand}(0,1)*\text{pbes}t_{\mathrm{kj}}}{\text{Nneigh}}\\ i\in \{0,1,\ldots ,n-1\},j\in \{0,1,\ldots ,d-1\},k\neq i \end{array}$$

where *N_neigh_* is the number of far neighbors of the i^th^ bee, and pbest is the best position that each bee found so far. *N_neigh_* of the i^th^ bee is computed using the Hamming distance equation for each bee to all of its neighbor bees.

Conversely, the onlooker bee phase focuses on local exploitation by updating food source positions based on information from the best near neighbor:


$$\begin{array}{c} v_{\mathrm{ij}}(t+1)=x_{\mathrm{ij}}(t)+\varphi (x_{\mathrm{ij}}(t)-x_{\mathrm{kj}}^{\text{best}})\\ i\in \{0,1,\ldots ,n-1\},j\in \{0,1,\ldots ,d-1\}\;\; \end{array}$$

To map these continuous updates into the binary feature space, a specialized probabilistic transfer function, *S*(*a_ij_*), was employed:


S(aij)=(1−exp(−ACit∗T))+∣tanh(aij)∣

or


$$a_{\mathrm{ij}}=\varphi (x_{\mathrm{ij}}-\mathrm{AP}B_{\mathrm{ij}})$$

$$\begin{array}{c} a_{\mathrm{ij}}=\varphi (x_{\mathrm{ij}}-x_{\mathrm{kj}}^{\text{best}})\\ i,k\in \{0,1,\ldots ,n-1\},i\neq k,j\in \{0,1,\ldots ,d-1\} \end{array}$$

where *a_ij_* represents the probability of a bit flip, *AC_i_* is the abandonment counter for the i^th^ solution, and *T* is a temperature-like scaling factor. If a randomly generated number is less than *S*(*a_ij_*), the binary bit is complemented (flipped); otherwise, the bit remains unchanged. The scout bee phase was triggered when a food source exceeded a limit of 10 iterations without improvement, at which point it was re-initialized randomly to prevent stagnation in local optima.

#### 2.5.3 Two-stage feature and sensor optimization using BMNABC

To transition from a theoretical model to a practical BCI application, it is essential to minimize system complexity without compromising performance. We implemented a two-stage optimization framework. Stage 1 focuses on reducing the dimensionality of the feature space, while Stage 2 focuses on the physical reduction of hardware (electrodes/optodes) to enhance user comfort and system portability.

##### 2.5.3.1 Stage 1: feature prioritization and reduction

**Figure 3:**
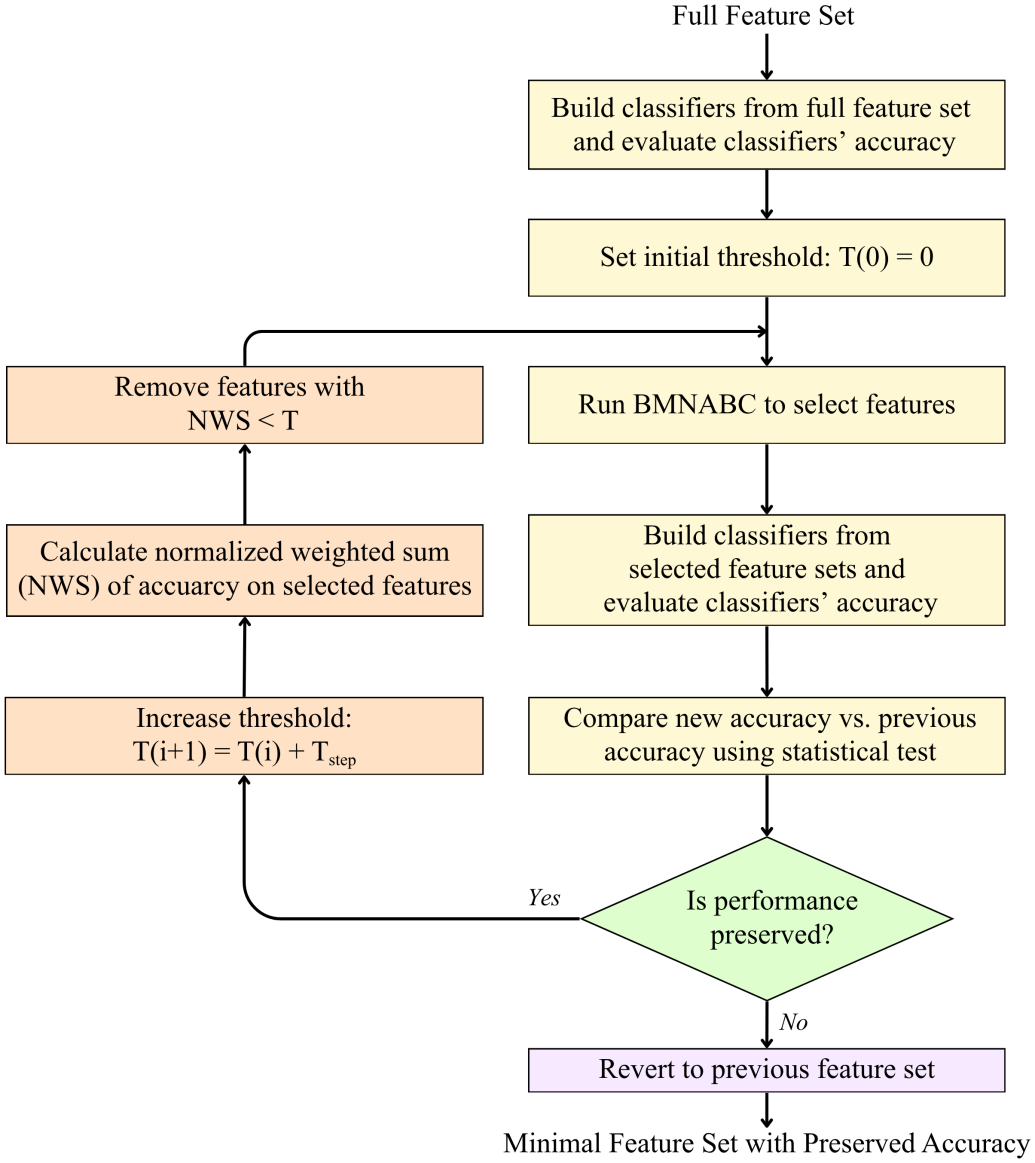
Flowchart of the Stage 1 feature prioritization and reduction process. The diagram illustrates the iterative loop where features are ranked using the normalized weighted sum (NWS) of classification accuracies. Features falling below an incremental threshold (T) are removed, and the BMNABC is re-executed. A statistical validation step ensures that feature removal does not significantly compromise performance before the threshold is increased further.

To achieve a minimal and practical sensor configuration suitable for BCI applications, we implemented an iterative feature reduction and validation process in our framework (as illustrated in Figure 3). This framework systematically identifies and removes the least informative features while ensuring that classification performance remains statistically indistinguishable from the optimal baseline. The process begins by establishing a performance baseline using the full feature set, with the feature removal threshold (*T*) initialized at zero.

The core of this stage is an iterative feedback loop designed to progressively refine the feature space. In each cycle, the BMNABC algorithm is executed on the current search space to identify optimal feature subsets. To maximize the robustness of the heuristic search, the BMNABC algorithm is executed for a defined number of independent runs (*N*_run_). In each run, the feature set selected by the BMNABC was evaluated by building a classification model using a selected classifier appropriate for the BCI task. The resulting classification accuracy served directly as the fitness value guiding the BMNABC’s search. This process yielded *N*_run_ feature subsets.

Following the search, a statistical validation step (e.g., the Mann–Whitney U test) is performed to compare the performance of the newly selected feature set against that of the previous valid set (or the full set for the initial iteration). This statistical test acts as a gatekeeper, determining whether the reduction in features has compromised the system’s efficacy. If the statistical analysis confirms that the classification performance is preserved—meaning there is no significant difference between the new and previous models—the framework proceeds to further reduce the feature space. It is important to note that these tests serve strictly as a screening mechanism rather than a final determination of feature significance. The robustness of the resulting feature set is subsequently validated by the BMNABC metaheuristic search, which evaluates the synergistic predictive power of features in combination.

The removal threshold is incremented by a defined step size (*T*_new_ = *T*_old_ + *T*_step_). The *N*_run_ feature sets are then aggregated, weighted, and normalized to assess the overall importance of each feature. The normalized weighted sum (NWS) value is calculated for each feature, quantifying its collective contribution to the final accuracy across all successful BMNABC trials. Any feature with an NWS value falling below this threshold is deemed non-contributory and is permanently removed from the BMNABC search space for subsequent iterations. The choice of step size should be considered to balance computational efficiency and search granularity. In our preliminary evaluations, we found that 0.1 was fine-grained enough to capture the necessary shifts in the feature-performance trade-off without the excessive computational overhead that smaller increments would require. This ensured the search remained efficient while still identifying an optimal feature subset.

Conversely, if the statistical test indicates a significant performance degradation, the loop terminates immediately. The system then reverts to the feature set from the previous successful iteration, yielding a final output that represents the most efficient, minimal feature configuration without compromising classification efficacy.

##### 2.5.3.2 Stage 2: sensor optimization via recursive backward elimination

**Figure 4:**
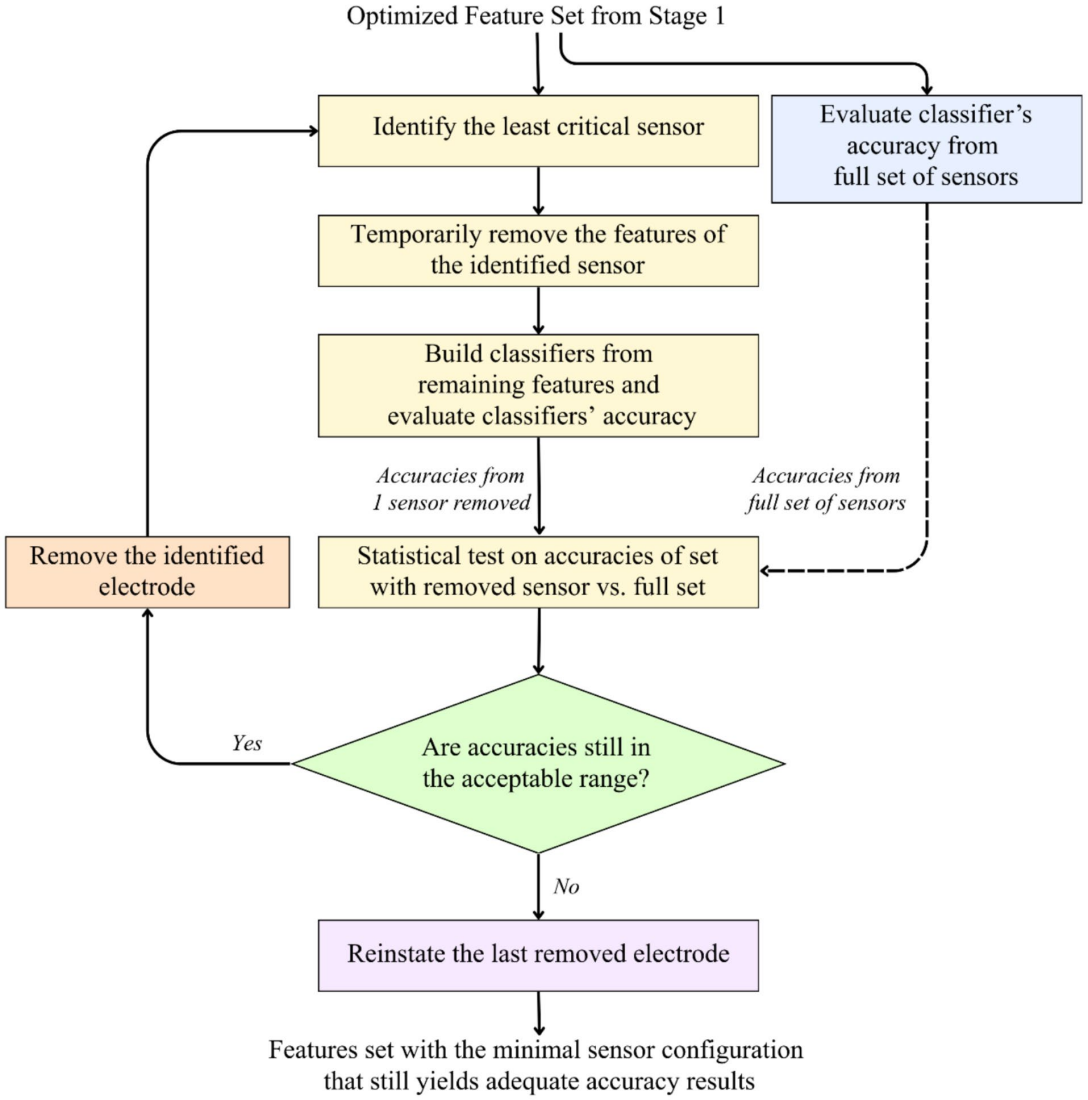
Flowchart of the Stage 2 sensor optimization process. This diagram illustrates the recursive backward elimination (RBE) strategy used to identify the minimal set of EEG electrodes and fNIRS optodes. The process involves iteratively removing the least critical sensor based on median accuracy until performance drops below an acceptable threshold.

The second stage of optimization aims to identify the minimal subset of sensors (EEG electrodes and fNIRS optodes) required to detect implicit learning accurately. Reducing the number of physical sensors lessens the physical burden on the user and decreases the computational complexity of the analysis pipeline, making the system less resource-intensive. To achieve this, a Recursive Backward Elimination (RBE) strategy was implemented, as illustrated in Figure 4. This procedure operates on the refined feature set output from Stage 1.

The process commences by establishing a performance baseline using the optimized feature set obtained from Stage 1. Classifiers are trained and evaluated using this full sensor configuration to generate a distribution of accuracy scores. This baseline distribution serves as the reference standard against which all reduced configurations are statistically compared. Following the baseline assessment, an iterative elimination cycle is initiated to prune the sensor array. In each iteration, the system identifies the “least critical” sensor currently remaining in the set by pinpointing the sensor whose contribution to the model’s classification accuracy is minimal. The features associated with this identified sensor are temporarily removed, and new classification models are built using the remaining features. Their accuracy is then evaluated to generate a new performance distribution.

Subsequently, a statistical validation test is performed to compare the accuracy distribution of the sensor-depleted set against the baseline accuracy of the full sensor set. This comparison determines whether the removal has caused a statistically significant degradation in performance. If the statistical test indicates that the accuracies remain within an acceptable range—meaning performance is maintained or improved—the identified sensor is permanently removed from the configuration, and the process iterates to find the next candidate for removal. Conversely, if the accuracies drop below the acceptable tolerance, indicating that the removed sensor was essential for model efficacy, the loop terminates. The last removed sensor is then reinstated to ensure the final model retains its predictive power. The resulting configuration is the most streamlined sensor set, representing the minimum hardware requirements necessary to yield acceptable classification performance.

The final output of this comprehensive framework is a streamlined, highly discriminative feature set associated with a minimal configuration of EEG electrodes and fNIRS optodes. These outputs serve a dual purpose. From a neuroscientific perspective, the selected features act as specific biomarkers, isolating the precise frequency bands and cortical regions—such as the prefrontal cortex—that are critical for the emergence of implicit learning. From an engineering perspective, the reduction in physical sensors and data dimensionality significantly lowers computational overhead and enhances user comfort. This establishes a practical foundation for developing wearable, real-time BCI systems capable of monitoring learning states in naturalistic environments.

## 3 Results and discussion

The primary objective of this study was to develop and validate a feature selection framework capable of accurately identifying implicit learning events from simultaneously acquired EEG and fNIRS data. To demonstrate the framework’s efficacy in addressing the challenges of high-dimensional multimodal data, we structured the evaluation into three distinct phases. First, we evaluate the performance of the proposed multimodal fusion strategy by comparing it against unimodal (EEG-only and fNIRS-only) approaches. This comparison is essential to verify the hypothesis that integrating electrophysiological and hemodynamic signals yields superior detection accuracy. Second, we analyze the outcomes of the Stage 1 feature prioritization, validating the framework’s ability to extract meaningful neural signatures from the complex feature space. Finally, we assess the results of the Stage 2 sensor optimization, investigating the trade-off between hardware reduction and classification accuracy. This final analysis aims to demonstrate the feasibility of a streamlined, resource-efficient BCI configuration that maintains high performance with a minimal sensor configuration, thereby advancing the development of practical, real-world applications.

### 3.1 Performance comparison of unimodal and multimodal feature sets

We aimed to empirically validate the hypothesis that fusing EEG and fNIRS data at the feature level yields superior classification performance compared to using either modality in isolation. In addition, we also aimed to assess the efficacy of the BMNABC algorithm in navigating the complex search spaces of unimodal versus multimodal datasets. To achieve this, feature vectors were constructed for three distinct experimental conditions: (1) EEG-only, utilizing Multiscale Entropy features; (2) fNIRS-only, utilizing Wavelet Transform reconstructed features; and (3) Multimodal, constructed by concatenating the EEG and fNIRS feature vectors.

The BMNABC feature selection process was executed 30 times independently for each condition across a suite of classifiers (including Random Forests, k-Nearest Neighbors, SVM variants, and Decision Trees). The control parameters for the BMNABC algorithm were initialized as follows: Population Size = 20, Maximum Cycle Number = 100, and the Limit parameter (controlling the abandonment of a food source) = 10. To establish a rigorous baseline comparison between unimodal and multimodal feature sets, the classification accuracy using the full feature set (without selection) was also computed. For the performance evaluation, the “Best Food Source” (optimal feature subset) identified in each run was validated using 5-fold cross-validation to maximize data utility for the preliminary comparative study. To ensure statistical stability, 30 independent repetitions were performed, with the dataset being re-randomized before each repetition. For a fair comparison, all three conditions were processed using an identical validation strategy.

**Figure 5:**
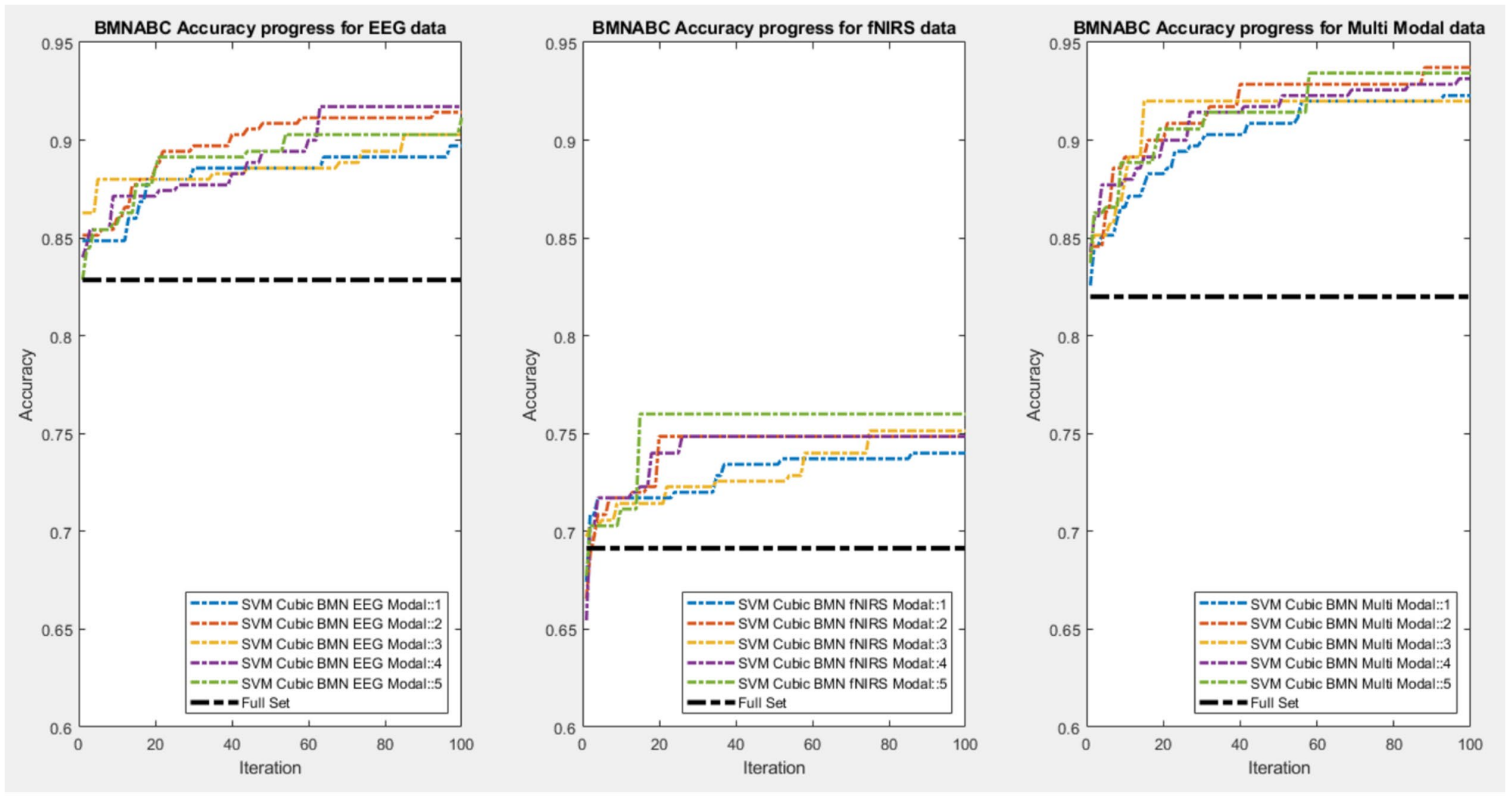
Convergence analysis of the BMNABC algorithm using the SVM cubic classifier. The three panels display the “best-so-far” accuracy over iterations for (left) EEG-only, (center) fNIRS-only, and (right) multimodal datasets. Colored dashed lines represent independent experimental runs, while the black dash-dot line indicates the baseline median accuracy using the full feature set without selection.

Figures 5 illustrate the optimization trajectories for the SVM classifier with cubic kernel as an example. The plots display the “Best-So-Far” accuracy over iterations for representative runs. The convergence behavior of the BMNABC algorithm provides insight into its optimization capability. As observed in the figure, the Multimodal data (Right Panel) consistently achieves higher convergence points (reaching >90% accuracy) compared to the EEG-only (Left Panel) and fNIRS-only (Center Panel) datasets. Furthermore, the BMNABC algorithm successfully improves accuracy significantly beyond the Full Feature Set baseline (represented by the black dash-dot line) in all cases. This demonstrates the critical role of the BMNABC feature selection in removing noise and redundant information from high-dimensional biological data to uncover the underlying learning patterns.

The results indicate that the multimodal approach significantly outperforms unimodal EEG and fNIRS configurations across all tested classifiers (detailed rankings provided in Supplementary Tables S1, S2). To quantitatively evaluate the benefits of data fusion, we identified the five highest-performing classifiers from our initial screening. Table 1 summarizes the mean classification accuracies (± standard deviation) for these top five models across the 30 independent runs. The results demonstrate a clear performance hierarchy. The Multimodal configuration consistently outperformed both unimodal setups. For instance, robust classifiers such as SVM Cubic and SVM Gaussian achieved accuracies exceeding 91 and 94% respectively, with the multimodal approach, representing a substantial gain over the fNIRS-only baseline (~68–71%) and a statistically significant improvement over the EEG-only baseline (~89–90%). Statistical significance was verified using a Kruskal-Wallis test, which indicated significant differences among the three data types (*p* < 0.005) for all classifiers. Subsequent pairwise comparisons using the Mann–Whitney U test confirmed that the Multimodal performance was significantly superior (*p* < 0.05) to both EEG and fNIRS individual modalities in the vast majority of cases. Detailed statistical rankings and pairwise *p*-values for all tested classifiers are provided in Supplementary Tables S1, S2.

**Table 1:** Summary of mean classification accuracies (±SD) for the top five performing classifiers across 30 independent runs.

Classifier	EEG-only	fNIRS only	Multimodal EEG-fNIRS
SVM gaussian	0.8917 ± 0.0110	0.6792 ± 0.0229	0.9430 ± 0.0053
SVM cubic	0.9003 ± 0.0104	0.7182 ± 0.0075	0.9184 ± 0.0106
SVM polynomial	0.8812 ± 0.0138	0.7596 ± 0.0094	0.9152 ± 0.0062
Random forest	0.8669 ± 0.0073	0.7440 ± 0.0060	0.9043 ± 0.0068
kNN cosine	0.8649 ± 0.0156	0.7459 ± 0.0055	0.9042 ± 0.0061

The empirical results strongly support the proposed feature-level fusion framework. The significant performance boost observed in the Multimodal setup confirms that EEG and fNIRS provide complementary information: EEG captures rapid neural dynamics (temporal resolution), while fNIRS captures hemodynamic responses (spatial specificity). Our findings align with previous studies advocating for feature-level integration, such as Perpetuini et al. (2020), where combining data at the feature level consistently improved performance. This stands in contrast to some decision-level fusion approaches (e.g., Ahn et al., 2016), where high correlation between classifier outputs can limit the benefits of fusion. By fusing at the feature level and applying BMNABC, our model was able to exploit the non-linear interactions between electrophysiological and hemodynamic signals, identifying a “hidden” structure in the data that neither modality could fully capture alone. This confirms that for implicit learning detection—a complex cognitive process involving multiple brain regions—a multimodal approach is not just beneficial, but essential.

### 3.2 Evaluation of stage 1: feature prioritization and identification of neural correlates

While the results in Section 3.1 confirmed that fusing EEG and fNIRS data significantly enhances classification accuracy, they treat the classification model largely as a “black box.” To advance our understanding of implicit learning, it is crucial to look inside this box and identify which neural signals drive this performance. Therefore, the primary purpose of this evaluation was to validate the efficacy of the Stage 1 feature prioritization process. By analyzing the features that survived the rigorous selection process, we aim to isolate specific neural “biomarkers”—defined by electrode locations, frequency bands, and hemodynamic responses—that are most critical for identifying implicit learning events.

As detailed in the methodology (Section 2.5.3.1), this identification was achieved using the NWS metric. We selected three distinct algorithms that demonstrated superior stability in our preliminary screenings: k-Nearest Neighbors with Cosine similarity (kNNCosine), Support Vector Machine with Gaussian Kernel (SVMGaussian), and Support Vector Machine with Polynomial Kernel (SVMPoly). For each classifier, BMNABC feature selection was performed over 30 independent runs (Nrun = 30), with the dataset re-randomized for each run to account for the stochastic nature of metaheuristic searches and to generate a robust pool of “Best Position” feature sets. Consistent with the configuration in the previous phase, the BMNABC algorithm was initialized with a population size of 20 bees, a maximum cycle number of 100, and a limit parameter of 10 for abandoning exhausted food sources. 5-fold cross-validation was used to evaluate classification accuracy, which served as the fitness values for BMNABC. Each run yielded an optimized ‘best position’ representing a specific feature set.

The NWS was calculated for each classifier independently to quantify the collective contribution of features specific to that model’s decision boundary. The iterative reduction process utilized an incremental step size of 0.1. To ensure a rigorous statistical comparison at each threshold step, rather than relying on a single performance metric, each of the 30 resulting feature sets was subjected to a hold-out validation strategy repeated 100 times, with full re-randomization of the training and testing splits for each evaluation. This process generated a distribution of 3,000 accuracy scores for each condition, providing the statistical power necessary for the Mann–Whitney U test to detect any significant degradation in performance caused by the iterative feature removal. This approach effectively captures data variability and ensures that the selected biomarkers possess genuine discriminative power across diverse data partitions, mitigating the risk of selection bias.

The iterative validation process demonstrated that the BMNABC framework successfully reduced feature dimensionality across all tested architectures, though the optimal stopping points varied slightly depending on the classifier’s specific decision boundary requirements. For the kNNCosine classifier, the optimal balance between feature reduction and accuracy preservation was achieved at a threshold of *T* = 0.2. In contrast, the SVMPoly classifier allowed for a more aggressive reduction, maintaining statistical stability up to a threshold of *T* = 0.4.

Tables 2, 3 illustrate the resulting NWS heatmaps for the kNNCosine and SVMPoly classifiers, respectively. These tables highlight the physiological features most critical for each specific model. The distinct separation between high-NWS features and suppressed features (blackout cells) confirms that the framework can effectively isolate spatially and spectrally localized signals relevant to implicit learning, regardless of the classifier used.

**Table 2:** Normalized weighted sum of features for the kNNCosine classifier at threshold *T* = 0.2.

Feature	AF3	AF4	F3	F4	F5	F6	F7	F8	FZ
Gamma	0.553	0.525	0.758	0.467	0.759	0.234	0.496	0.260	0.409
Beta	0.642	0.758	0.408	0.232	0.380	0.758	0.263	0.612	0.701
Alpha	0.525		0.351		0.118	0.116	0.320	0.525	0.174
Theta	0.235	0.350	0.233	0.379	0.350	0.116		0.175	0.874
Delta	0.524	0.205		0.641	0.350	0.175	0.496	0.525	0.553
L5	0.728	0.203	0.232	0.350	0.146	0.175	0.670	0.117	
L6	0.816			0.116	0.116	0.233	0.874	0.495	

**Table 3:** Normalized weighted sum of features for the SVMPoly classifier at threshold *T* = 0.4.

Feature	AF3	AF4	F3	F4	F5	F6	F7	F8	FZ
Gamma	0.813	0.842	0.902			0.723			
Beta	0.751	0.812			0.692	0.812		0.572	0.902
Alpha							0.633	0.843	0.571
Theta		0.783	0.843						0.631
Delta	0.783	0.662		0.663	0.782			0.723	0.573
L5	0.812						0.783		
L6	0.813						0.843		

To provide a coherent neurophysiological interpretation, we focus our analysis on the features identified by the SVMPoly configuration (Table 3), as this model achieved the highest degree of feature reduction (*T* = 0.4) while maintaining robust classification performance. The feature selection process identified a cluster of significant electrodes in the frontal region. Specifically, AF3, AF4, and F3 were prominent in the Gamma band, while AF4 and F6 were significant in the Beta band. Additionally, AF3 and F5 were identified in the Delta band. These findings align strongly with existing neuroscientific literature. The identified electrodes (AF3, AF4, F3, F4, F6) spatially correspond to the Dorsolateral Prefrontal Cortex (DLPFC), approximating Brodmann Area 9 (BA9). This region is well-documented to contribute to higher cognitive functions essential for learning, including working memory, planning, abstract reasoning, and organization (Raye et al., 2002). Furthermore, the specific dominance of Gamma and Beta oscillations coincides with the findings of Rose et al. (2010), who established that increased activity in these high-frequency bands is a hallmark of implicit learning processes, reflecting the intense cognitive integration required during the acquisition of hidden patterns.

A critical insight provided by the multimodal approach is the specific contribution of fNIRS signals. The Stage 1 evaluation highlighted the F7 optode location (specifically Wavelet levels L5 and L6) as a statistically significant feature (see Table 3). The F7 location maps to Brodmann Area 47 (BA47), located in the Inferior Frontal Gyrus. This region has been implicated in the processing of syntax in oral and sign languages, musical syntax, and the semantic aspects of language (Raye et al., 2002). This finding is particularly notable given that implicit learning paradigms—including the one used in this study—often rely on Artificial Grammar Learning (AGL) or sequence prediction tasks that mimic grammatical structures. The identification of BA47 confirms that the fNIRS signals successfully captured the hemodynamic correlates of syntactic processing that EEG alone might miss or spatially blur. This reaffirms our hypothesis that implicit learning engages specific language-processing networks and that fNIRS provides complementary “hidden” information essential for robust event detection.

### 3.3 Evaluation of stage 2: sensor optimization for streamlined BCI applications

The final phase of this study addressed the practical challenges of BCI deployment. While Stage 1 identified critical feature vectors, the physical acquisition of these signals still required a full montage of electrodes and optodes. To mitigate the burden of cumbersome monitoring devices and reduce computational complexity for real-time applications, the purpose of this evaluation was to identify the minimal subset of physical sensors required to maintain high classification accuracy.

Our feature prioritization analysis revealed that fNIRS signals provide unique, complementary information, specifically capturing hemodynamic responses in the Inferior Frontal Gyrus (BA47) associated with syntactic processing. Since this hemodynamic “hidden channel” is essential for robustly detecting implicit learning events, the engineering challenge shifted to streamlining the accompanying EEG array. The goal was to reduce the high-density EEG setup to its most essential components, thereby creating a lightweight hybrid system that retains the complementary strengths of both modalities.

The evaluation employed the Recursive Backward Elimination (RBE) strategy described in Section 2.5.3.2. We established a performance baseline using the optimized feature set from Stage 1 on the complete nine-electrode configuration. To ensure the reliability of this baseline, we utilized a rigorous statistical protocol: each classifier model (kNNCosine, SVMGaussian, and SVMPoly) was subjected to 30 independent training iterations, with each iteration evaluated 100 times. The median of the resulting 3,000 accuracy scores served as the robust performance metric. Starting from the full set, sensors were iteratively removed based on their contribution to the model’s accuracy. The elimination process continued until the classification accuracy dropped below a predefined acceptability threshold of 85%. This threshold was selected to ensure that the streamlined system remains reliable enough for practical implicit learning detection while maximizing portability.

**Figure 6:**
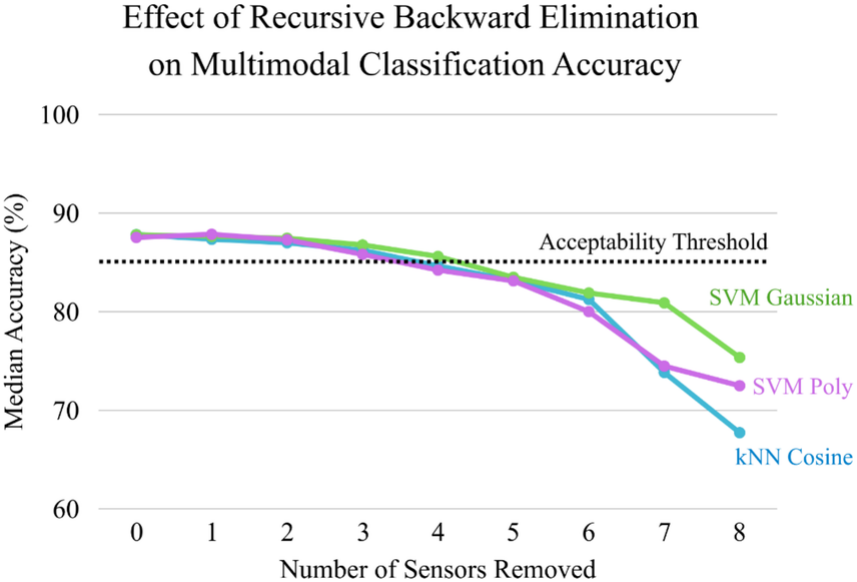
Performance degradation analysis during iterative sensor optimization. The graph illustrates the median classification accuracy across 3,000 evaluations for the three top-performing classifiers (kNNCosine, SVMGaussian, and SVMPoly) as sensors are systematically removed from the configuration. The horizontal dashed line represents the predefined 85% acceptability threshold.

The baseline evaluation of the three classifier setups utilizing the complete sensor set revealed a consistent median accuracy ranging between 87 and 88%. This high baseline confirms that the features selected in Stage 1 were robust across different classifier architectures. The RBE process revealed a clear distinction between redundant and critical sensors. As illustrated in the performance degradation analysis (Figure 6), the systematic removal of electrodes F4, F5, and F8 resulted in a negligible impact on classification accuracy across all three classifiers, with accuracies remaining well above 85%. Removing these three sensors caused the median accuracy to fluctuate by less than 1–2%, suggesting their limited contribution to the discrimination of “Fast” versus “Slow” learner events in this multimodal paradigm.

However, further analysis of the performance degradation curve in Figure 6 reveals an even more streamlined possibility for highly resource-constrained applications. The threshold can be determined using an ‘elbow point’ criterion, in which the objective is to identify the most parsimonious feature set that maintains statistical parity with the full-dimensional model. The elbow point is a critical task in machine learning and system design for identifying optimal trade-offs between cost and performance (Antunes et al., 2025). A performance drop was considered acceptable only if it was not statistically significant (Mann–Whitney U, *p* > 0.05) and did not exceed a predefined margin of 5%. The framework allows for an aggressive reduction of six electrodes, leaving a minimal array of only three critical sensors: FZ, F7, and AF3. Further feature removal would cause a disproportionate drop in predictive power. In this extreme reduction scenario, the classification accuracy remains statistically viable for basic event identification, resulting in a 66% reduction from the original EEG montage and significantly lowering the barrier for portable, real-world deployment.

The retention of AF3, F7, and FZ as the “essential core” in both scenarios provides profound neurophysiological validation of our methodology. Specifically, the persistence of AF3 (DLPFC) as the final EEG sensor to be retained confirms that executive control and working memory functions localized in the dorsolateral prefrontal cortex are the primary electrophysiological drivers for identifying implicit learning. Similarly, the critical nature of the F7 (BA47) location reinforces the importance of syntactic and semantic pattern processing, while FZ (Midline Frontal) likely captures integrated activity from the anterior cingulate and medial prefrontal regions involved in error monitoring during the learning process. Moreover, fNIRS provides spatially precise hemodynamic data from BA47, ensuring that the system remains accurate even when the EEG spatial resolution is drastically reduced. From an engineering perspective, these findings confirm that a hybrid BCI utilizing a sparse EEG array coupled with targeted fNIRS optodes is the most practical architecture for wearable, real-time monitoring of learning states in naturalistic environments.

### 3.4 Limitations

While the proposed BMNABC-based framework demonstrates high efficacy in identifying implicit learning events, several limitations must be addressed to contextualize the current findings:

- *Sample size and demographic diversity*: This study involved a dataset of 30 participants, with 9 achieving the implicit learning criterion. We acknowledge that this small sample size and the narrow demographic—consisting exclusively of college students aged 21–29—represent significant constraints on the study’s scope. In this section, we discuss these constraints and frame the current study as a proof-of-concept for the BMNABC-based optimization framework. We emphasize that although the current results are statistically valid for this specific group, future research with larger, more diverse cohorts—including different age ranges and cognitive profiles—is necessary to confirm broader generalizability.
- *Paradigm and task specificity*: The results presented are derived from a single cognitive paradigm. While the framework successfully identified key neural biomarkers in the prefrontal cortex (BA9 and BA47), further investigation is required to determine if these signatures and the resulting reduced sensor sets remain stable across different types of implicit learning tasks or alternative recording sessions.
- *Computational and real-time considerations*: The iterative heuristic search performed by the BMNABC algorithm is computationally intensive, which may pose challenges for immediate, per-session calibration. Future research should focus on optimizing search efficiency and exploring cross-subject transfer learning to facilitate the development of “plug-and-play” systems suitable for real-time, closed-loop applications in naturalistic environments.

## 4 Conclusion

This study successfully developed and validated a two-stage feature selection and sensor optimization framework that systematically bridges the gap between high-dimensional multimodal data and practical hardware constraints, utilizing implicit learning event detection as a primary use case. By integrating EEG and fNIRS data through a BMNABC-driven optimization process, we demonstrated that the proposed multimodal framework significantly enhances classification performance, achieving accuracies exceeding 94%. We successfully identified a highly reduced subset of neurophysiological features and sensor locations, primarily centered in the dorsolateral prefrontal cortex (BA9) and the inferior frontal gyrus (BA47), that maintain high classification accuracy. While applied here to implicit learning, the framework is designed to be classifier-agnostic and application-independent, providing a robust methodology for identifying critical neural signatures and streamlining hardware requirements in a wide array of BCI applications.

The primary contribution of this work is the demonstration that metaheuristic optimization can effectively handle the high dimensionality of multimodal neuroimaging data, significantly reducing the hardware requirements for implicit learning detection. While these results provide robust proof of concept for the proposed framework within a controlled demographic, we acknowledge that the current findings are specific to the paradigm employed. Future research is essential to validate the stability of these biomarkers across more diverse populations and a range of cognitive tasks. Ultimately, this work provides a methodological foundation for developing more practical, wearable, and efficient BCI systems to monitor non-conscious cognitive processes.

## Data Availability

The original contributions presented in the study are included in the article/Supplementary material, further inquiries can be directed to the corresponding author.
