# Supplementary material for: Toward practical BCIs: a BMNABC-based feature selection and sensor optimization framework for implicit learning detection from multimodal EEG-fNIRS data
Source: Front Hum Neurosci. 2026 May 4;20:1778884. doi: 10.3389/fnhum.2026.1778884 (PMC13180941; doi:10.3389/fnhum.2026.1778884)
Supplement: Supplementary file 1 [file Table_1.docx]

Supplementary Material

**Table S1**. Kruskal-Wallis test results comparing the distribution of classification accuracies across three data modalities (EEG, fNIRS, and Multimodal) for all evaluated classifiers.

| **Classifier** | **Datatype** | **N** | **Accuracy (Mean±SD)** | **Mean Rank** | **DF** | **H** | **p-value** |
| --- | --- | --- | --- | --- | --- | --- | --- |
| Random Forest | EEG | 30 | 0.8669±0.0073 | 45 | 2 | 10.5 | 0.005 |
|  | fNIRS | 30 | 0.7440±0.0060 | 15 |  |  |  |
|  | **Multimodal** | **30** | **0.9043±0.0068** | **60** |  |  |  |
| Decision Tree Fine | EEG | 30 | 0.7850±0.0124 | 40 | 2 | 12.5 | 0.002 |
|  | fNIRS | 30 | 0.6718±0.0103 | 15 |  |  |  |
|  | **Multimodal** | **30** | **0.8231±0.0086** | **65** |  |  |  |
| Decision Tree Med | EEG | 30 | 0.7932±0.0158 | 40 | 2 | 12.5 | 0.002 |
|  | fNIRS | 30 | 0.6878±0.0082 | 15 |  |  |  |
|  | **Multimodal** | **30** | **0.8232±0.0140** | **65** |  |  |  |
| Decision Tree Coarse | EEG | 30 | 0.7613±0.0082 | 38 | 2 | 11.58 | 0.003 |
|  | fNIRS | 30 | 0.6807±0.0080 | 17 |  |  |  |
|  | **Multimodal** | **30** | **0.7615±0.0110** | **65** |  |  |  |
| kNN Medium | EEG | 30 | 0.8881±0.0101 | 40.5 | 2 | 12.2 | 0.002 |
|  | fNIRS | 30 | 0.7529±0.0093 | 15 |  |  |  |
|  | **Multimodal** | **30** | **0.8974±0.0068** | **64.5** |  |  |  |
| kNN Coarse | EEG | 30 | 0.8941±0.0056 | 41.5 | 2 | 11.8 | 0.003 |
|  | fNIRS | 30 | 0.7527±0.0073 | 15 |  |  |  |
|  | **Multimodal** | **30** | **0.8998±0.0095** | **63.5** |  |  |  |
| kNN Cosine | EEG | 30 | 0.8649±0.0156 | 40 | 2 | 12.5 | 0.002 |
|  | fNIRS | 30 | 0.7459±0.0055 | 15 |  |  |  |
|  | **Multimodal** | **30** | **0.9042±0.0061** | **65** |  |  |  |
| SVM Linear | EEG | 30 | 0.8086±0.0058 | 40 | 2 | 12.5 | 0.002 |
|  | fNIRS | 30 | 0.6081±0.0115 | 15 |  |  |  |
|  | **Multimodal** | **30** | **0.8161±0.0131** | **65** |  |  |  |
| SVM Polynomial | EEG | 30 | 0.8812±0.0138 | 40 | 2 | 12.5 | 0.002 |
|  | fNIRS | 30 | 0.7596±0.0094 | 15 |  |  |  |
|  | **Multimodal** | **30** | **0.9152±0.0062** | **65** |  |  |  |
| SVM Cubic | EEG | 30 | 0.9003±0.0104 | 40 | 2 | 12.5 | 0.002 |
|  | fNIRS | 30 | 0.7182±0.0075 | 15 |  |  |  |
|  | **Multimodal** | **30** | **0.9184±0.0106** | **65** |  |  |  |
| SVM Gaussian | EEG | 30 | 0.8917±0.0110 | 40 | 2 | 12.5 | 0.002 |
|  | fNIRS | 30 | 0.6792±0.0229 | 15 |  |  |  |
|  | **Multimodal** | **30** | **0.9430±0.0053** | **65** |  |  |  |
| kNN Fine | EEG | 30 | 0.8828±0.0059 | 44 | 2 | 10.82 | 0.004 |
|  | fNIRS | 30 | 0.7473±0.0100 | 15 |  |  |  |
|  | **Multimodal** | **30** | **0.8927±0.0151** | **61** |  |  |  |

**Table S2** Mann-Whitney U test results for pairwise comparisons of classification accuracies between the Multimodal and individual unimodal feature sets (Multimodal vs. EEG and Multimodal vs. fNIRS).

| **Mann-Whitney U Test** | | **Mean Accuracy** | | | **N** | **DF** | **z score** | **p value** |
| --- | --- | --- | --- | --- | --- | --- | --- | --- |
|  |  | **Multi** | **EEG** | **fNIRS** |  |  |  |  |
| Random Forest | Multi vs EEG | 0.904 | 0.866 |  | 30 | 29 | -2.611 | 0.009 |
|  | Multi vs fNIRS | 0.904 |  | 0.744 | 30 | 29 | -2.611 | 0.009 |
| D Tree Fine | Multi vs EEG | 0.823 | 0.785 |  | 30 | 29 | -2.611 | 0.009 |
|  | Multi vs fNIRS | 0.823 | 0 | 0.671 | 30 | 29 | -2.611 | 0.009 |
| D Tree Medium | Multi vs EEG | 0.823 | 0.793 |  | 30 | 29 | -2.611 | 0.009 |
|  | Multi vs fNIRS | 0.823 |  | 0.687 | 30 | 29 | -2.611 | 0.009 |
| D Tree Coarse | Multi vs EEG | 0.761 | 0.761 |  | 30 | 29 | -2.611 | 0.009 |
|  | Multi vs fNIRS | 0.761 |  | 0.680 | 30 | 29 | -2.611 | 0.009 |
| kNN Fine | Multi vs EEG | 0.892 | 0.882 |  | 30 | 29 | -1.985 | 0.047 |
|  | Multi vs fNIRS | 0.892 |  | 0.747 | 30 | 29 | -2.611 | 0.009 |
| kNN Medium | Multi vs EEG | 0.897 | 0.888 |  | 30 | 29 | -2.507 | 0.012 |
|  | Multi vs fNIRS | 0.897 |  | 0.752 | 30 | 29 | -2.611 | 0.009 |
| kNN Coarse | Multi vs EEG | 0.899 | 0.894 |  | 30 | 29 | -2.978 | 0.022 |
|  | Multi vs fNIRS | 0.899 |  | 0.752 | 30 | 29 | -2.611 | 0.009 |
| kNN Cosine | Multi vs EEG | 0.904 | 0.864 |  | 30 | 29 | -2.611 | 0.009 |
|  | Multi vs fNIRS | 0.904 |  | 0.745 | 30 | 29 | -2.611 | 0.009 |
| SVM Linear | Multi vs EEG | 0.816 | 0.808 |  | 30 | 29 | -2.611 | 0.009 |
|  | Multi vs fNIRS | 0.816 |  | 0.608 | 30 | 29 | -2.611 | 0.009 |
| SVM Poly | Multi vs EEG | 0.915 | 0.881 |  | 30 | 29 | -2.611 | 0.009 |
|  | Multi vs fNIRS | 0.915 |  | 0.759 | 30 | 29 | -2.611 | 0.009 |
| SVM Cubic | Multi vs EEG | 0.918 | 0.900 |  | 30 | 29 | -2.611 | 0.009 |
|  | Multi vs fNIRS | 0.918 |  | 0.718 | 30 | 29 | -2.611 | 0.009 |
| SVM Gaussian | Multi vs EEG | 0.943 | 0.891 |  | 30 | 29 | -2.611 | 0.009 |
|  | Multi vs fNIRS | 0.943 |  | 0.679 | 30 | 29 | -2.611 | 0.009 |
